# *Atp13a5* Marker Reveals Pericyte Specification in the Mouse Central Nervous System

**DOI:** 10.1523/JNEUROSCI.0727-24.2024

**Published:** 2024-09-11

**Authors:** Xinying Guo, Shangzhou Xia, Tenghuan Ge, Yangtao Lin, Shirley Hu, Haijian Wu, Xiaochun Xie, Bangyan Zhang, Sonia Zhang, Jianxiong Zeng, Jian-Fu Chen, Axel Montagne, Fan Gao, Qingyi Ma, Zhen Zhao

**Affiliations:** ^1^Center for Neurodegeneration and Regeneration, Zilkha Neurogenetic Institute and Department of Physiology and Neuroscience, Keck School of Medicine, University of Southern California, Los Angeles, California 90033; ^2^Department of Anesthesiology, Guangzhou Women and Children’s Medical Center, Guangzhou Medical University, Guangdong Provincial Clinical Research Center for Child Health, Guangzhou 510623, China; ^3^Department of Basic Sciences, The Lawrence D. Longo, MD Center for Perinatal Biology, Loma Linda University School of Medicine, Loma Linda, California 92350; ^4^Department of Neurosurgery, Second Affiliated Hospital, School of Medicine, Zhejiang University, Hangzhou 310009, China; ^5^Songjiang Research Institute, Songjiang Hospital, Shanghai Jiao Tong University School of Medicine, Shanghai 201600, China; ^6^Center for Craniofacial Molecular Biology, University of Southern California, Los Angeles, California 90033; ^7^Centre for Clinical Brain Sciences, UK Dementia Research Institute at University of Edinburgh, Edinburgh EH16 4SB, United Kingdom; ^8^Caltech Bioinformatics Resource Center, California Institute of Technology, Pasadena, California 91125

**Keywords:** blood–brain barrier, development, mouse model, mural cells, pericyte, single-cell transcriptomics

## Abstract

Perivascular mural cells including vascular smooth cells (VSMCs) and pericytes are integral components of the vascular system. In the central nervous system (CNS), pericytes are also indispensable for the blood–brain barrier (BBB), blood–spinal cord barrier, and blood–retinal barrier and play key roles in maintaining cerebrovascular and neuronal functions. However, the functional specifications of pericytes between CNS and peripheral organs have not been resolved at the genetic and molecular levels. Hence, the generation of reliable CNS pericyte-specific models and genetic tools remains very challenging. Here, we report a new CNS pericyte marker in mice. This putative cation-transporting ATPase 13A5 (*Atp13a5*) marker was identified through single-cell transcriptomics, based on its specificity to brain pericytes. We further generated a knock-in model with both tdTomato reporter and Cre recombinase. Using this model to trace the distribution of *Atp13a5-*positive pericytes in mice, we found that the tdTomato reporter reliably labels the CNS pericytes, including the ones in spinal cord and retina but not peripheral organs. Interestingly, brain pericytes are likely shaped by the developing neural environment, as *Atp13a5-*positive pericytes start to appear around murine embryonic day 15 (E15) and expand along the cerebrovasculature. Thus, *Atp13a5* is a specific marker of CNS pericyte lineage, and this *Atp13a5-*based model is a reliable tool to explore the heterogeneity of pericytes and BBB functions in health and diseases.

## Significance Statement

Pericyte is a key component of the blood–brain barrier (BBB) and highly implicated in neurological and neurodegenerative diseases. However, current genetic tools for brain pericytes often come with limitations, due to the lack of specificity to the pericytes in the brain or central nervous system (CNS), as well as the overlap with other cell types, particularly vascular smooth muscle cells. Here, we identified that *Atp13a5* is a CNS-specific pericyte marker based on mouse single-cell transcriptomics and further validate it using a knock-in model carrying *Atp13a5*-driven tdTomato reporter and Cre recombinase. The success of the *Atp13a5*-based model opens new possibility of genetic manipulations targeting only CNS pericytes in vivo and studying their biology and functions in health and diseases more specifically.

## Introduction

Pericytes are vascular mural cells that play key roles in vascular development and the maintenance of microvascular functions (Armulik et al., 2011; Sweeney et al., 2016). They cover microvessels including precapillary arterioles, capillaries, and postcapillary venules, while vascular smooth muscle cells (VSMCs) occupy large-diameter arteries and veins (Sweeney et al., 2016). In the central nervous system (CNS), pericytes are vital integrators of neurovascular functions (Nikolakopoulou et al., 2019; Hartmann et al., 2021) and indispensable for a functional blood–brain barrier (BBB; Armulik et al., 2010; Bell et al., 2010). Based on early genetic lineage tracing studies, it has been proposed that brain pericytes may originate from neural crest cells, while the peripheral ones mainly arise from the mesothelium (Armulik et al., 2011; Yamazaki and Mukouyama, 2018). However, no genetic marker has been identified so far for a clear classification of pericytes between CNS and peripheral organs, which also become a major hurdle for genetic manipulations and lineage tracing of CNS pericytes (Sweeney et al., 2016).

Various genetic markers of pericytes have been tested in the past decade. Platelet-derived growth factor receptor beta (PDGFRβ) is one of the most well-known molecular markers for pericytes, as PDGF-B/PDGFRβ signaling is essential for its fate determination (Armulik et al., 2010; Bell et al., 2010). In addition, chondroitin sulfate proteoglycan 4 (*CSPG4*), desmin, vimentin, regulator of G-protein signaling 5 (*RGS5*), and CD13/aminopeptidase N (*APN*) are also broadly used to label pericytes in research. As these markers are often shared with VSMCs, fibroblasts or oligodendrocyte precursor cells (OPCs), transgenic mouse models based on these alleles, including *Cspg4-Cre* and *Cspg4-dsRed* (Zhu et al., 2008), *Pdgfrb-EGFP* and *Pdgfrb-Cre* (Gerl et al., 2015; Jung et al., 2018), and *Rgs5-EGFP* (Nisancioglu et al., 2008), have limitations when applied to brain pericytes. New pericyte models based on *Abcc9* (ATP Binding Cassette Subfamily C Member 9) and the inwardly rectifying potassium channel *Kcnj8* were developed more recently (Ando et al., 2022), showing impressive specificity to pericytes over VSMCs in the brain. However, none of these markers can differentiate CNS pericytes from the peripheral ones. Recently, we used both *Pdgfrb* and *Cspg4* promoters to control Cre recombinase expression (Nikolakopoulou et al., 2019). This model exhibits restricted Cre activity in pericytes compared with VSMCs, but minimal expression in peripheral organs remains (Nikolakopoulou et al., 2019), and the sophisticated design with two promoters and two recombinases (Cre and Flp) also confines its applications.

To address this gap, we compiled multiple mouse transcriptomic datasets and identified that *Atp13a5* is much more specific to CNS pericytes than other current markers. *Atp13a5* encodes a member of the P5 subfamily of P-type ATPases and is predicted to be a cation transporter (Sørensen et al., 2010). Next, we generated an *Atp13a5-2A-CreERT2-IRES-tdTomato* knock-in model, by replacing the endogenous stop codon with a cassette with a self-cleaving 2A peptide sequence and an internal ribosome entry site (IRES) for cistronic expression of both Cre recombinase and tdTomato reporter. We then characterized the distribution of *Atp13a5-*driven tdTomato reporter and validated the CreER recombinase activity and confirmed that this genetic tool based on *Atp13a5* is successful and can be utilized for genetic manipulations of CNS pericytes in vivo. Profiles of the tdTomato reporter in this new model are on a par with the bioinformatic results, as they are only found in the CNS and colocalized exclusively with CD13^+^ pericyte profiles. Moreover, using this reporter mice, we found that *Atp13a5* is also developmentally regulated, and the specialization of brain pericytes coincided with BBB establishment during embryonic development. Therefore, our findings demonstrate that *Atp13a5-2A-CreERT2-IRES-tdTomato* knock-in model is a useful tool to study brain pericyte biology associated with BBB development or CNS diseases.

## Materials and Methods

### Animals

Mice were housed in plastic cages on a 12 h light/dark cycle with access to water *ad libitum* and a standard laboratory diet. All procedures were approved by the Institutional Animal Care and Use Committee at the University of Southern California and followed National Institutes of Health guidelines. All animals were included in the study. All animals were randomized for their genotype information. All experiments were carried out blind: the operators responsible for the experimental procedures and data analysis were blinded and unaware of group allocation throughout the experiments. For all experiments, male and female animals were used, and no apparent sex difference was observed.

### Generation of the Atp13a5-2A-CreERT2-IRES-tdTomato knock-in model

To generate *Atp13a5-CreERT2-tdTomato knock-in mouse*, donor DNA templates encoding self-cleaving 2A peptide, CreERT2, internal ribosome entry site, tdTomato, and flp recombinase (*2A-CreERT2-Frt-IRES-tdTomato-Frt*) were synthesized. These sequences were flanked by 1,184 bp sequences and 1,237 bp sequences homologous to the last exon and 3′ UTR region of *Atp13a5* gene. In addition, the IRES-tdTomato sequence is further flanked by two flp recombinase target (frt) sites. Next, these donor vector containing the *2A-CreERT2-Frt-IRES-tdTomato-Frt* cassette and gRNA (matching forward strand of gene: TTTTGGACTAGACTGTAACCAGG) were coinjected into fertilized C57BL/6N mouse eggs to generate targeted conditional knock-in offspring. The F0 founder animals were genotyped by PCR and sequence analysis, and three F1 mice were generated and further confirmed with Southern blotting for both 5′ arm and 3′ arm insertion sequences. Tamoxifen (Sigma, T-5648) administration in *Atp13a5-2A-CreERT2-IRES-tdTomato; Ai162* mice were performed intraperitoneally at 40 mg/kg per day for four or seven consecutive days, as we described previously (Nikolakopoulou et al., 2019). The line is currently maintained as homozygous.

### Bioinformatics

#### scRNA-seq data for mouse brain vasculature and multiple organs

For scRNA-seq dataset for mouse brain vasculature, we obtained the cell count matrix from Gene Expression Omnibus (GEO) with the series record GSE98816 and GSE99058 (Vanlandewijck et al., 2018). The data represent the expression levels of 18,435 genes in 3,186 cells. The mouse brain tissue was harvested for Smart-seq2, and sequencing was performed on a HiSeq2500 at the National Genomics Infrastructure (NGI), Science for Life Laboratory, Sweden, with single 50 bp reads (dual indexing reads). For scRNA-seq dataset for postnatal development, we obtained the cell count matrix from GEO with the series record GSE104323 (Hochgerner et al., 2018). The data represent the expression levels of 27,933 genes in 24,185 cells. The dentate gyrus from different ages was microdissected. All cDNA synthesis, library preparation, and sequencing were carried out as instructed by the manufacturer (10x Genomics Chromium Single Cell Kit Version 2). Libraries were sequenced on an Illumina HiSeq 4000.

#### scRNA-seq data preprocessing

The data processing of the scRNA-seq data were performed with the Seurat Package (v.3.1.5) in R (v.3.6.2; Butler et al., 2018; Stuart et al., 2019). The basic scRNA-seq analysis was run using the pipeline provided by Seurat Tutorial (https://satijalab.org/seurat/v3.0/immune_alignment.html) as of June 24, 2019. In general, we set up the Seurat objects from different groups in experiments for normalizing the count data present in the assay. This achieves log-normalization of all datasets with a size factor of 10,000 transcript per cell. For different Seurat objects, FindVariableFeatures() function was used to identify outlier genes on a “mean variability plot” for each object. The nFeatures parameter is 2,000 as the default for the selection method called “vst.” These resulted genes serve to illustrate priority for further analysis.

#### Data processing

The dataset on all cells were used to scale and center the genes. Firstly, principal component analysis (PCA) was used for linear dimensionality reduction with default computes the top 30 principal components. By applying the JackStraw() function, JackStrawPlot() function, and ElbowPlot() function, we identified the principal components for further analysis. Then, PCA results were used as the input for the Uniform Manifold Approximation and Projection (UMAP) dimensional reduction.

We identified clusters of cells by a shared nearest neighbor (SNN) modularity optimization-based clustering algorithm. The algorithm first calculated k-nearest neighbors and computed the k-NN graph and then optimizes the modularity function to determine clusters.

#### Determination of cell-type identity

To determine the cell type, we used FindAllMarkers() function with parameters min.pct and thresh.use set to 0.25 to find markers in each cluster and known marker genes that have been previously reported (Saunders et al., 2018) to determine cell-type identity. These include, but are not limited to, *Snap25* for neuron, *Cldn10* for astrocyte, *Mbp* for oligodendrocyte, *Cldn5* for EC, *Kcnj8* for PC, *Acta2* for VSMC, *Ctss* for microglial, and *Col1a1* for fibroblast-like cell.

### Cellular biology-related procedures

#### Fluorescence in situ hybridization

Fluorescence in situ hybridization (FISH) was performed using the RNAscope technology (Advanced Cell Diagnostics). Tissue sample preparation and pretreatment were performed on fixed brains cut into 15 µm sections mounted onto SuperFrost Plus glass slides following the manufacturer's protocol (ACD documents 323100). After dehydration and pretreatment, slides were subjected to RNAscope Multiplex Fluorescent Assay (ACD documents 323100). RNAscope probes for mouse *Atp13a5*, positive control, and negative control were hybridized for 2 h at 40°C in the HybEZ Oven and the remainder of the assay protocol was implements. Subsequently, the slides were subjected to immunohistochemistry. The fluorescent signal emanating from RNA probes and antibodies was visualized and captured using a Nikon AIR MP+ confocal/multiphoton microscope (Nikon). All FISH images presented were projection of 10-image stacks (0.5 µm intervals) obtained from the cerebral cortex, and a standard smoothing step was applied during image postprocessing (Nikon NIS-Elements Software).

#### Chromogenic in situ hybridization

Chromogenic in situ hybridization was performed using the RNAscope technology (Advanced Cell Diagnostics). Tissue sample preparation and pretreatment were performed on FFPE brain samples cut into 10 µm sections mounted onto SuperFrost Plus glass slides following the manufacturer's protocol (ACD documents 322452). After deparaffinization and pretreatment, slides were subjected to RNAscope chromogenic ISH-Red Assay (ACD documents 322360). RNAscope probes for mouse *Atp13a5*, positive control, and negative control were hybridized for 2 h at 40°C in the HybEZ Oven and the remainder of the assay protocol was implements. The *Atp13a5* Red signal was examined under a standard bright-field microscope.

#### Immunohistochemistry

Animals were anesthetized and perfused, and brains were removed and postfixed as we described previously (Nikolakopoulou et al., 2019). The brain, spinal cord, kidney, liver, and heart tissue were also collected, postfixed, and cut at 35 µm thickness using a vibratome (Leica). After that, sections were blocked with 5% normal donkey serum (Vector Laboratories) and 0.1% Triton X-100 in 0.01 M PBS and incubated with primary antibodies diluted in blocking solution overnight at 4°C. The primary antibody information is the following: Goat anti-mouse aminopeptidase N/ANPEP (CD13; R&D Systems; AF2335; 1:100), Rat anti-mouse vascular adhesion molecule (VCAM1; MilliporeSigma; CBL1300; 1:200), Mouse anti-α-smooth muscle actin (SMA, MilliporeSigma; A5228, 1:200), Rabbit anti-mouse ionized calcium binding adaptor molecule 1 (Iba-1; Wako, 019-19741; 1:200), Rabbit anti-mouse NeuN (Millipore, ABN78, 1:500), and Rabbit anti-mouse Olig2 (Millipore; AB9610; 1:200). To visualize brain microvessels, sections were incubated with DyLight 488 or 649-conjugated *L. esculentum* lectin as we have described previously (Nikolakopoulou et al., 2019). After incubation with primary antibodies, sections were washed with PBS for three times and incubated with fluorophore-conjugated secondary antibodies. Sections were imaged with a Nikon AIR MP+ confocal/multiphoton microscope (Nikon). *Z*-stack projections and pseudocoloring were performed using Nikon NIS-Elements Software. Image postanalysis was performed using ImageJ software. In Figure 3*D*, the percentage of *Atp13a5*-tdTomato^+^ cells located on VCAM1-positive venules, αSMA-positive arterioles, or capillaries were normalized to total tdTomato cells quantified in each mouse.

#### DNA isolation and genotyping

Mouse genomic DNA was isolated from tail biopsies (2–5 mm) and following overnight digestion at 56°C into 100 μl of tail digestion buffer containing 10 mM Tris-HCl, pH 9.0, 50 mM KCl, 0.1% Triton X-100, and 0.4 mg/ml Proteinase K. Next, the tail will be incubated at 98°C for 13 min to denature the Proteinase K. After centrifugation at 12,000 rpm for 15 min, the supernatants were collected for PCR. Wild-type primers (432 bp): forward: 5′- CAGTTTCACTCTCATCTCCCTTG -3′; reverse: 5′-CTGCAAGGCTCGGTATGTTGAAGTG -3′. Knock-in primers (212 bp): forward: 5′- CACCTGTTCCTGTACGGCAT - 3′; reverse: 5′- CTGCAAGGCTCGGTATGTTGAAGTG-3′. The PCR conditions were as follows: (1) 94°C for 3 min; (2) 35 cycles at 94°C for 30 s, 60°C for 30 s, and 72°C for 35 s; and (3) 72°C for 5 min. PCR products were separated on 2% agarose gel.

#### RNA isolation and real-time quantitative PCR

The mouse brains were harvested and frozen in dry ice and store at −80°C. Total RNA was isolated using Quick-RNA Miniprep Kit (ZYMO research, R1054) according to the manufacturer's instructions, and 10 μl of RNA was used for real-time quantitative PCR using qScript One-Step SYBR Green qRT-PCR kit (Quantabio, 95087) according to the manufacturer's instructions. *Gapdh* was used as an internal control for normalization. The PCR conditions were as follows: 50°C for 5 min, 95°C for 30 s, and followed by 40 cycles at 95°C for 3 s and 60°C for 25 s. The primer information is listed in Table 1.

#### Quantification and statistical analysis

Sample sizes were calculated using nQUERY, assuming a two-side alpha-level of 0.05, 80% power, and homogeneous variances for the two samples to be compared, with the means and SEM for different parameters predicted from pilot study. All the data are presented as mean ± SEM as indicated in the figure legends and were analyzed by GraphPad Prism 8. For multiple comparisons, Bartlett's test for equal variances was used to determine the variances between the multiple groups, and one-way analysis of variance (ANOVA) followed by Tukey’s test was used to test statistical significance, using GraphPad Prism 8 software. A *p* value of <0.05 was considered statistically significant.

**Table 1.** Key resources table

Reagent or resource	Source	Identifier
Genomic datasets		
Mouse brain vasculature	Gene Expression Omnibus	GSE98816
		GSE99058
Postnatal development	Gene Expression Omnibus	GSE104323
Single-cell RNA-sequencing data processing		
Seurat Package (v.3.1.5)	R	v.3.6.2
FindVariableFeatures function	R	v.3.6.2
FindAllMarkers function	R	v.3.6.2
FindMarkers function	R	v.3.6.2
Principal component analysis (PCA)	R	v.3.6.2
Uniform Manifold Approximation and Projection (UMAP)	R	v.3.6.2
Shared Nearest Neighbor (SNN) clustering	R	v.3.6.2
Experimental Models: Organisms/Strains		
Atp13a5-CreERT2-tdTomato	This study	
Pdgfrb-EGFP	GENSAT	JN169
Ai162	Jackson Laboratory	031562
DNA isolation and genotyping		
GoTaq Green Master Mix	Promega	M7122
Wild-type ATP13a5 forward primer	CAGTTTCACTCTCATCTCCCTTG	
Wild-type ATP13a5 reverse primer	CTGCAAGGCTCGGTATGTTGAAGTG	
Atp13a5-CreERT2-tdTomato forward primer	CACCTGTTCCTGTACGGCAT	
Atp13a5-CreERT2-tdTomato reverse primer	CTGCAAGGCTCGGTATGTTGAAGTG	
RNA Isolation and RT qPCR		
Quick-RNA Miniprep Kit	ZYMO Research	R1054
qScript One-Step SYBR Green qRT-PCR kit	Quantabio	95087
Atp13a5 forward primer	GAGGTGTTTGGCTACCATACC	
Atp13a5 reverse primer	GGGATGCAACTGGTCCACA	
Gapdh forward primer	AGGTCGGTGTGAACGGATTTG	
Gapdh reverse primer	TGTAGACCATGTAGTTGAGGTCA	
Antibodies		
Goat polyclonal anti-CD13	R&D Systems	AF2335
Rat monoclonal anti-VCAM1	Millipore Sigma	CBL1300
Mouse monoclonal anti-SMA	Millipore Sigma	A5228
Rabbit polyclonal anti-Iba-1	Wako	019-19741
Rabbit polyclonal anti-GFAP	Dako	z0334
Rabbit polyclonal anti-NeuN	Millipore Sigma	ABN78
Rabbit anti-mouse Olig2	Millipore Sigma	AB9610
DyLight 488-conjugated *L. esculentum* lectin	Thermo Fisher Scientific	L32470
DyLight 649-conjugated *L. esculentum* lectin	Thermo Fisher Scientific	L32472
Immunohistochemistry		
Vibratome	Leica	VT1200
Cryostat	Leica	CM3050 S
Normal Donkey Serum	Jackson ImmunoResearch	AB_2337258
In situ hybridization		
SuperFrost Plus Micro Slide	VWR	48311-703
Atp13a5 RNAscope probe	Advanced Cell Diagnostics	Catalog #417211
Positive control RNAscope probe	Advanced Cell Diagnostics	Catalog #320881
Negative control RNAscope probe	Advanced Cell Diagnostics	Catalog #320871
HybEZ Oven	Advanced Cell Diagnostics	
RNAscope 2.5 HD Reagent Kit-RED	Advanced Cell Diagnostics	322350
RNAscope Multiplex Fluorescent V2 Assay	Advanced Cell Diagnostics	323100
Nikon A1R MP+ confocal/multiphoton microscope	Nikon	Nikon A1R
Revolve 4 brightfield and fluorescence microscope	Echo	RVL-100-G
Software		
GraphPad Prism	GraphPad Software	GraphPad Prism 8
ImageJ	NIH	

Further information and request for resources and reagents should be directed to and will be fulfilled by Zhen Zhao (zzhao@usc.edu).

## Results

### *Atp13a5* is specifically expressed in mouse brain pericytes

**Figure 1.**
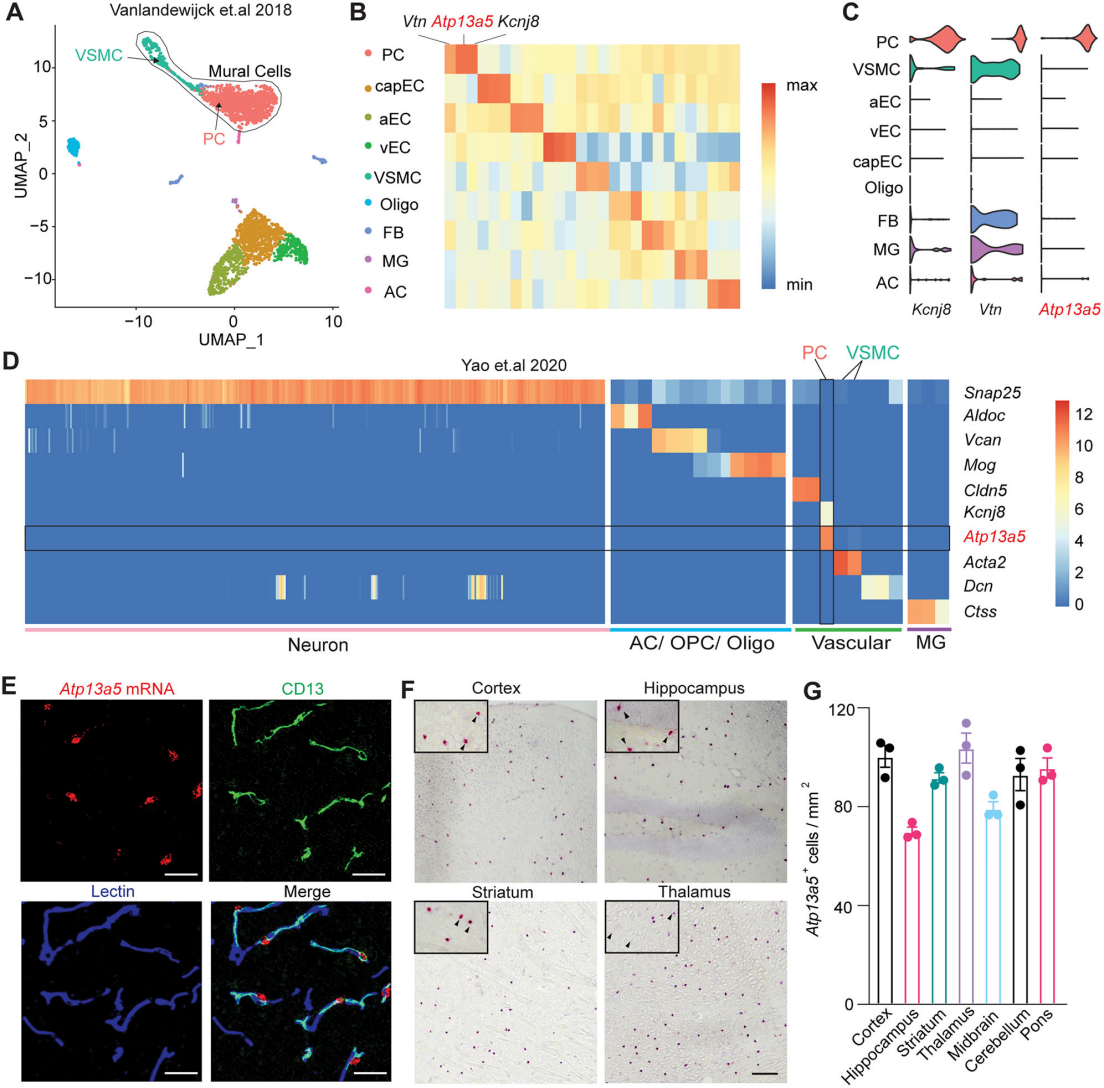
*Atp13a5* is specifically expressed by brain pericytes. ***A***, UMAP of brain vasculature transcriptomes. Mural cells are marked by black line. PC, pericytes; capEC, capillary endothelial cells; aEC, arterial endothelial cells; vEC, venous endothelial cells; VSMC, vascular smooth muscle cells; Oligo, oligodendrocytes; FB, fibroblast; MG, microglia; AC, astrocytes; OPC, oligodendrocyte progenitor cells. ***B***, Gene expression heatmap of the top 3 markers genes in each cluster. Pericyte markers: *Vtn*, *Atp13a5a*, and *Kcnj8*; capEC markers: *Slc7a5*, *Ctla2a*, and *Slc38a3*; aEC markers: *Bmx*, *Alpl*, and *Gkn3*; vEC markers: *Slc38a51*, *Vwf*, and *Flrt2*; VSMC markers: *Sncg*, *Pdlim3*, and *Gpr20*; Oligo markers: *Mbp*, *Cldn11*, and *Mag*; FB markers: *Lum*, *Col1a1*, and *Col6a1*; MG markers: *Trem2*, *Fcgr1*, and *AF251705*; AC markers: *Ntsr2*, *Mlc1*, and *Acsbg1*. Detailed gene lists are provided in Extended Data Figure 1-1 and Extended Data Tables 1-1 and 1-2. ***C***, Violin plots showing the distribution of expression level of the top 3 pericyte markers across all nine cell types. ***D***, Gene expression heatmap of representative genes in Allen Institute's dataset. ***E***, Representative images for *Atp13a5* mRNA expression (red) and immunostaining for CD13^+^ pericytes (green), and lectin^+^ endothelia cells (blue) in cortex. Scale bar, 50 µm. Sections: 15 µm thickness. ***F***, Representative images for *Atp13a5* mRNA expression (red) in various mouse brain regions. Scale bar, 100 µm. Sections: 10 µm thick. ***G***, Number of *Atp13a5*^+^ cells per mm^2^ in different mouse brain regions. *n* = 3 mice. Data are presented in mean ± SEM.

To identify a new marker for mouse brain pericytes, we compared five different transcriptomic datasets (Armulik et al., 2010; Daneman, 2010; He et al., 2016; Ximerakis et al., 2019; Song et al., 2020) and found that only 16 genes were commonly identified among these studies (Extended Data Fig. 1-1*A*, Extended Data Table 1-1). More importantly, we analyzed the single-cell RNA sequencing (scRNA-seq) data of brain vasculature (Vanlandewijck et al., 2018). In total, 3,186 single-cell transcriptomes were collected for the secondary analysis using a new R Seurat Package (Butler et al., 2018; Stuart et al., 2019). In the Uniform Manifold Approximation and Projection (UMAP), nine major cell types were separated into clusters, which include pericytes (PC), VSMCs, arterial endothelial cells (aEC), venous endothelial cells (vEC), capillary endothelial cells (capEC), oligodendrocytes (Oligo), fibroblast (FB), microglia (MG), and astrocytes (AC; Fig. 1*A*), based on specific genetic markers (Extended Data Fig. 1-1*B*, Extended Data Table 1-2). The top 3 gene markers that differentiate pericytes from other brain cell types are vitronectin (*Vtn*), *Atp13a5*, and *Kcnj8* (Fig. 1*B*). Between brain pericytes and VSMCs, *Atp13a5* showed a clear specificity to pericytes, while *Vtn* and *Kcnj8* as most pan-pericyte markers are present in a subset of VSMC and FB (Fig. 1C). This indicates that *Atp13a5* and *Abcc9* are the only known markers specific to brain pericytes over VSMC and FB (Ando et al., 2022).

10.1523/JNEUROSCI.0727-24.2024.f1-1 Figure 1-1 ***Atp13a5* expression in the mouse brain. (A**) Venn plot showing the overlaid genes between different datasets. **(B)** Violin plots showing gene markers that distinguish across vasculature cells. Genes are colored by cell types. PC: Pericytes; capEC: capillary endothelial cells; aEC: arterial endothelial cells; vEC: venous endothelial cells; VSMC: vascular smooth muscle cells; Oligo: Oligodendrocytes; FB: Fibroblast; MG: microglia; AC: astrocytes. **(C)** Representative images for *Atp13a5* mRNA expression (Red) in various mouse brain region. Scale bar: 100  µm. Sections: 10  µm thick. **(D)** ISH of *Atp13a5* mRNA expression in various mouse brain region from Allen Brain Atlas. (**E**) Raw expression value of *Atp13a5* mRNA expression in various mouse brain region. ISH expression data are from Allen Brain Atlas obtained from 56 days old adult male C57BL/6J mice (available from: http://mouse.brain-map.org). OLF, olfactory bulb; CTXsp, cortex subplate. (**F**) Bar plot showing *Atp13a5* expression pattern in NCBI dataset. Red bar indicated the brain tissue. L_int: large intestine; S_int: small intestine; MG: mammary gland. Download Figure 1-1, TIF file.

10.1523/JNEUROSCI.0727-24.2024.t1-1 Table 1-1 **Pericyte markers from different transcriptomic datasets.** The pericyte marker genes identified in the 5 studies (Armulik et al., 2010; Daneman, 2010; He et al., 2016; Ximerakis et al., 2019; Song et al., 2020) are listed under each tab, and the 16 common genes were listed in the last tab. Download Table 1-1, XLSX file.

10.1523/JNEUROSCI.0727-24.2024.t1-2 Table 1-2 **All annotated genes from the single cell transcriptomic dataset.** The table contains all the 6596 annotated genes, with their relative enrichment in different brain cell types indicated in column G. The top cell type specific gene markers were used for plotting heatmap in Fig 1B. Download Table 1-2, CSV file.

In addition, we examined a dataset of 1,093,785 cells from multiple cortical and hippocampal areas (Yao et al., 2021) and found that *Atp13a5* is indeed specific to brain pericytes (Fig. 1*D*). To further validate its transcripts in the brain, we used FISH with RNAscope probes and found that *Atp13a5* mRNA is colocalized exclusively with CD13-positive pericyte profiles in the cortex, but not with other cells including endothelial cells (Fig. 1*E*). RNAscope results also showed that *Atp13a5-*positive cells are detected throughout the brain, including the cortex, hippocampus, striatum, thalamus, midbrain, pons, and cerebellum (Fig. 1*F*,*G*; Extended Data Fig. 1-1*C*). These results match with the *Atp13a5* in situ hybridization data from the Allen Brain Atlas (Extended Data Fig. 1-1*D,E*). More importantly, *Atp13a5* expression in mice is more specific to the brain tissues than peripheral organs, based on tissue-specific RNA-seq data on NCBI (Extended Data Fig. 1-1*F*), indicating that *Atp13a5* is perhaps a brain pericyte-specific marker. This represents a significant advantage for establishing a model to manipulate only the brain pericytes, as other pericyte markers including *Abcc9* are also found in peripheral organs (Ando et al., 2022).

### Generation of Atp13a5-2A-CreERT2-IRES-tdTomato knock-in model

**Figure 2.**
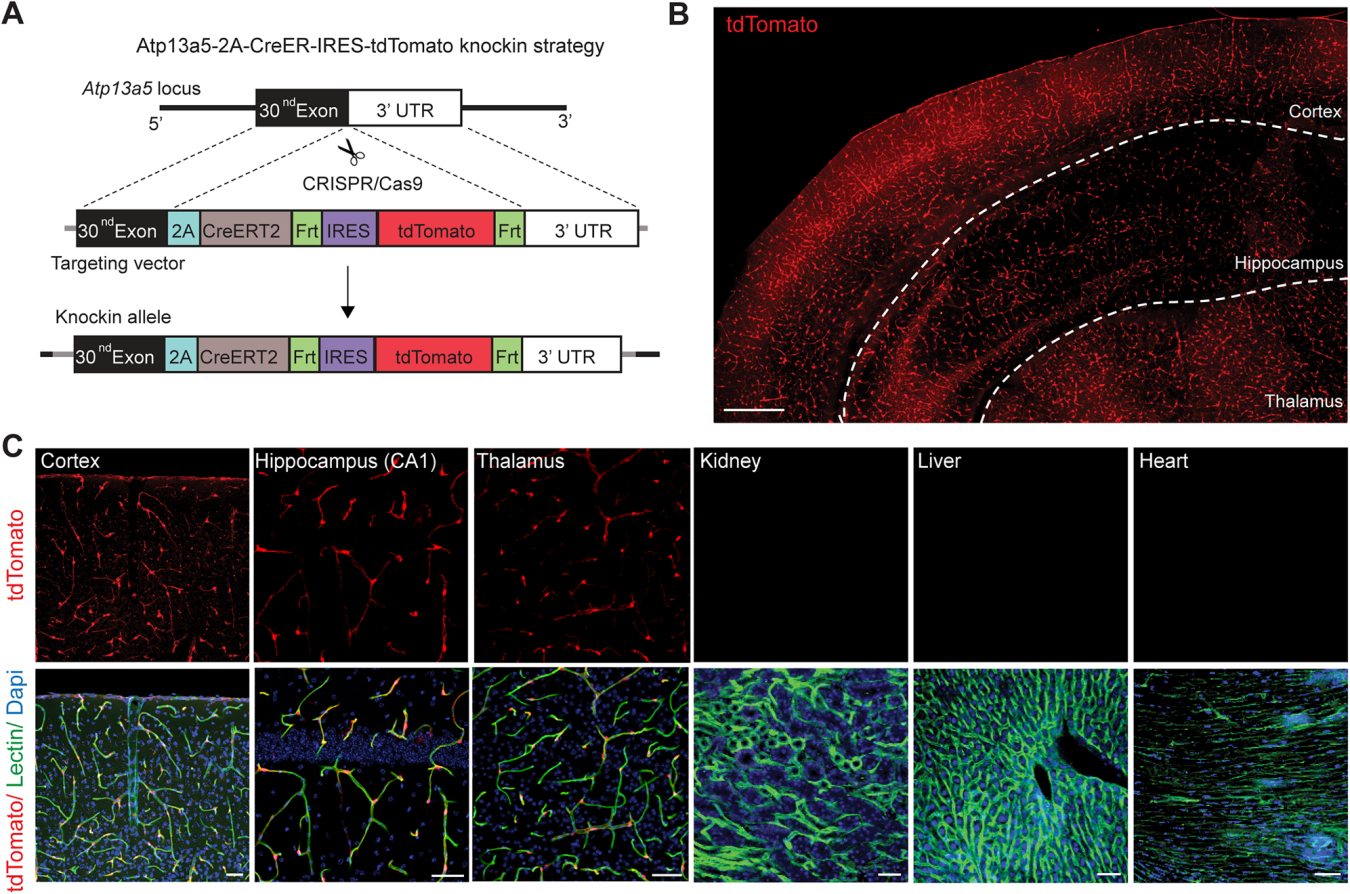
Generation and validation of the *Atp13a5-2A-CreERT2-IRES-tdTomato* model. ***A***, Schematic diagram showing the strategy for generating the *Atp13a5-2A-CreERT2-IRES-tdTomato* knock-in mice. See Materials and Methods for more details. ***B***, A representative tiled image of brain section from a heterozygous *Atp13a5-2A-CreERT2-IRES-tdTomato* mouse. Scale bar, 500 µm. ***C***, Representative confocal images of tdTomato, endothelial marker lectin, and Dapi in different tissues from a homozygous *Atp13a5-2A-CreERT2-IRES-tdTomato* mouse, including cortex, CA1 region of hippocampus, thalamus, kidney, liver, and heart (ventricular wall). Scale bar, 50 µm. Additional data are provided in Extended Data Figure 2-1.

Next, we generated a new transgenic model targeting the endogenous *Atp13a5* allele, to carry both Cre recombinase for genetic manipulation and a fluorescence reporter for imaging (Sjulson et al., 2016). More specifically, this *Atp13a5-2A-CreERT2-IRES-tdTomato* knock-in model harnesses the endogenous *Atp13a5* locus to drive the expression of both Cre and tdTomato, while preserving endogenous *Atp13a5* integrity by using the self-cleaving 2A peptide sequence (Tang et al., 2009) and an internal ribosome entry site (IRES; Hellen and Sarnow, 2001; Fig. 2*A*; also see Materials and Methods). One F0 founder was selected based on germline transmission and genome sequencing (Extended Data Fig. 2-1*A*); and the F1 generation was further tested with Southern blot analysis for the integrity of the knock-in allele (Extended Data Fig. 2-1*B*). Homozygous *Atp13a5-2A-CreERT2-IRES-tdTomato* (*Atp^tdT/tdT^*) mice are viable, appear normal, and are fertile. The knock-in cassette does not affect endogenous *Atp13a5* expression, as validated by quantitative real-time PCR (Extended Data Fig. 2-1*C,D*).

10.1523/JNEUROSCI.0727-24.2024.f2-1 Figure 2-1 **Generation and validation of the *Atp13a5-2A-CreERT2-IRES-tdTomato* model. (A)** Sequencing analysis of F1 *Atp^tdT/^*^+^ mice showing the insertions at 5’arm (top) and 3’arm (bottom). No additional mutation or deletion were found. **(B)** Southern blotting analysis showing F0 and 3 F1 founders (#1, #5, #7) carrying the intact allele based on hybridization of probes targeting the 5’ and 3’ arms, compared to a WT littermate. **(C)** Genotyping result showing the genotype of WT (with a 432-bp band), *Atp^tdT/^*^+^ (with 432-bp and 212-bp bands) and *Atp^tdT/tdT^* (with a 212-bp band). *Atp, Atp13a5; tdT, tdTomato*. **(D)** The endogenous gene expression of *Atp13a5* relative to *Gapdh* in brains from 8-week-old WT, *Atp^tdT/+^* and *Atp^tdT/tdT^* mice (n = 3 mice each). Data are presented in mean ± SEM. Download Figure 2-1, TIF file.

### *Atp13a5-tdTomato*-expressing cells are CNS pericytes

**Figure 3.**
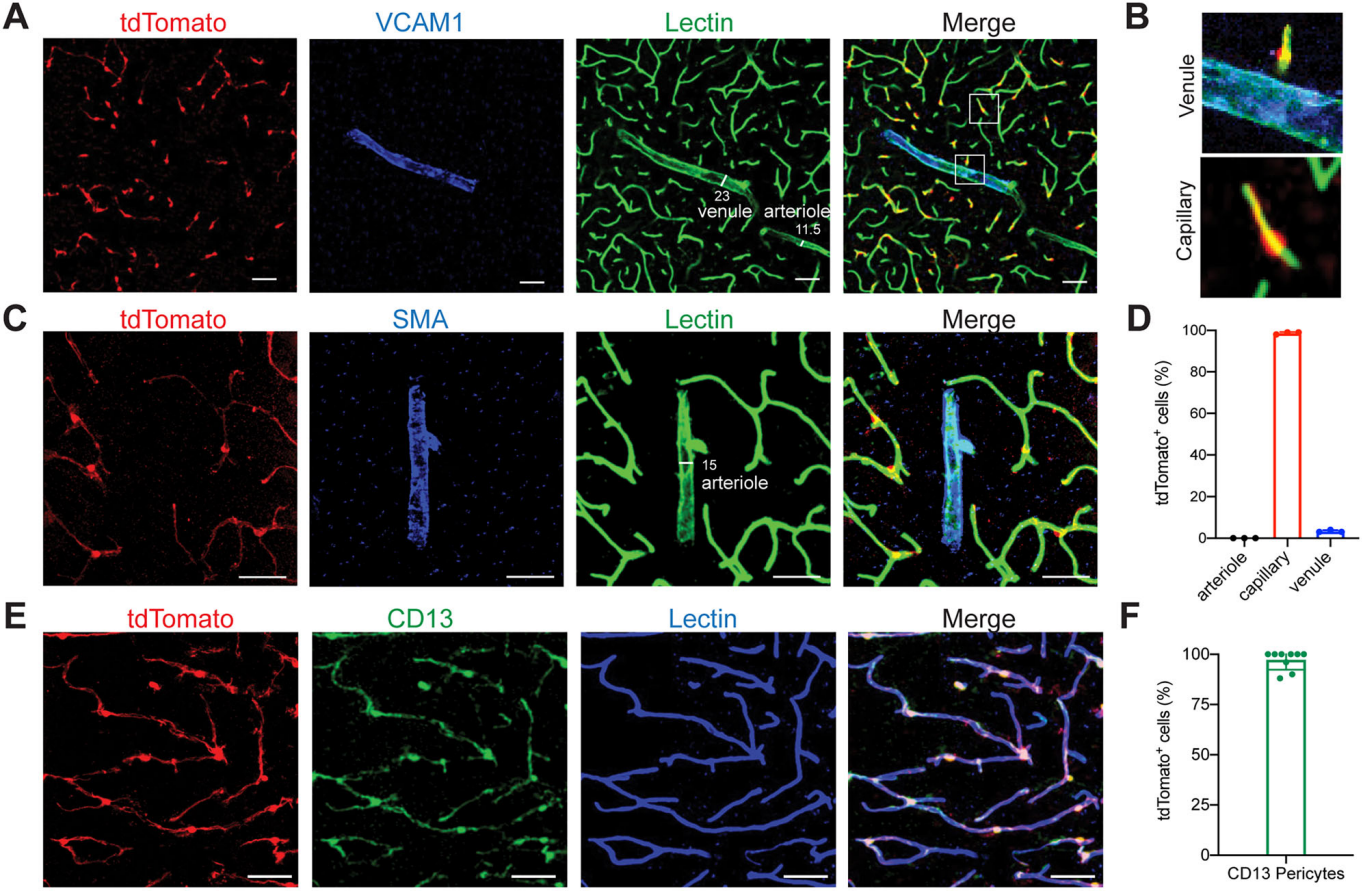
*Atp13a5-driven tdTomato* reporter expression in brain pericytes. ***A***, tdTomato expression on brain capillary of *Atp13a5-2A-CreERT2-IRES-tdTomato* knock-in mice, but not on VCAM1^+^ venules. Scale bar, 50 µm. ***B***, Representative images from the boxed regions in ***A***. ***C***, tdTomato expression on brain capillary, but not on SMA^+^ arterioles. Scale bar, 50 µm. ***D***, The percentage of tdTomato^+^ cells distributed among arterioles, capillaries, and venules in the cortex. Arteries and arterioles are identified by vessel diameter in combination with the presence of SMA. Veins and venules are identified by vessel diameter in combination with the presence of VCAM1 and the absence of SMA. Lectin^+^ vessels with diameters smaller than 6 µm are considered as capillaries. *n* = 3 mice. ***E***, Colocalization of tdTomato with pericyte marker CD13 (green) on lectin (blue) positive endothelium. Scale bar, 50 µm. ***F***, Quantification of the percentage of tdTomato^+^ cells in CD13^+^ pericytes. *n* = 9 mice. Data are presented in mean ± SEM. Additional data are provided in Extended Data Figures 3-1 and 3-2.

We found that *Atp13a5*-driven tdTomato is reliably expressed in adult heterozygous *Atp^tdT/+^* (Fig. 2*B*) and homozygous *Atp^tdT/tdT^* mice (Fig. 2*C*). The tdTomato profiles cover the lectin-labeled microvessels throughout the brain regions including the cortex, hippocampus, thalamus, striatum, midbrain, pons, and cerebellum, but are not seen in peripheral tissues such as the kidney, liver, or heart (Fig. 2*C*, Extended Data Fig. 2-1*E*). Additional immunostaining further confirmed that tdTomato is not expressed in large vessels, including VCAM1-positive venules (Fig. 3*A*,*B*) and smooth muscle cell actin (SMA)-positive arterioles (Fig. 3*C*,*D*). It is exclusively overlapped with CD13-positive pericytes in the brain (Fig. 3*E*,*F*). More importantly, no leakage of reporter expression was found in oligodendrocytes, neurons, microglia, or astrocytes (Extended Data Fig. 3-1*A–F*), further confirming its specificity. On the other hand, the currently widely used *Pdgfrb-EGFP* model labels pericytes in the brain and peripheral organs (Extended Data Fig. 3-2*A,B*). Therefore, the *Atp13a5-2A-CreERT2-IRES-tdTomato* model is the first genetic tool that targets brain pericytes specifically.

10.1523/JNEUROSCI.0727-24.2024.f3-1 Figure 3-1 **Characterization of *Atp13a5-2A-CreERT2-IRES-tdTomato* mouse brain.** (**A**) Representative images for *Atp13a5-*tdT reporter expression in mouse striatum, midbrain, pons and cerebellum regions. **(B-E)** Representative confocal images showing that tdTomato is not expressed in Olig2^+^ Oligodendrocytes (**B**), ionized calcium binding adaptor molecule 1 (Iba1)^+^ microglia (**C**), NeuN^+^ cortical neurons (**D**), and glial fibrillar acidic protein (GFAP)^+^ astrocytes (**E**). High magnification of boxed region in **E** is shown in **F**, and orthogonal view is shown in **G**. **A-E**: Scale bar: 50  µm. **F** and **G**: Scale bar: 25  µm. Download Figure 3-1, TIF file.

10.1523/JNEUROSCI.0727-24.2024.f3-2 Figure 3-2 **EGFP reporter expression in *Pdgfrb-EGFP* mouse. (A)** A representative image of *Pdgfrb-EGFP* mouse brain. Scale bar: 200  µm. Sections: 35  µm thick. **(B)** EGFP reporter expression in brain (cortex) and peripheral tissues such as kidney, liver and heart. Scale bar: 100  µm. Sections: 35  µm thick. (**C-E**) Characterization of *Atp13a5*-tdTomato; *Pdgfrb*-EGFP double reporter mice. (**C**) Representative confocal images of cortical section from *Atp13a5*-tdTomato; *Pdgfrb*-EGFP mice. Bar = 30  µm. (**D**) Quantification of diameters of capillary, postcapillary venules and precapillary arterioles with *Atp13a5*^tdT+^ and *Pdgfrb*^EGFP+^ pericytes, or *Atp13a5*^tdT-^ and *Pdgfrb*^EGFP+^ mural cells. (**E**) Representative confocal images of ovary section from *Atp13a5*-tdTomato; *Pdgfrb*-EGFP mice, using the same imaging setting as in **C**. Bar = 50  µm. Boxed region is re-scanned with increased exposure, and shown in the bottom. Download Figure 3-2, TIF file.

To better illustrate this, we crossed the *Atp13a5*-tdTomato model with *Pdgfrb*-EGFP mice (Extended Data Fig. 3-2*C*) and compared *Atp13a5*^tdT+^ and *Pdgfrb*^EGFP+^ pericytes with *Atp13a5*^tdT−^ and *Pdgfrb*^EGFP+^ mural cells on vessels with different diameters (Extended Data Fig. 3-2*D*). Capillaries were all covered by *Atp13a5*-tdTomato^+^ and *Pdgfrb*-EGFP^+^ pericytes. On postcapillary venules and precapillary arterioles, both *Atp13a5*^tdT+^ and *Pdgfrb*^EGFP+^ pericytes and *Atp13a5*^tdT−^ and *Pdgfrb*^EGFP+^ mural cells were found; however, *Atp13a5*^tdT+^ and *Pdgfrb*^EGFP+^ pericytes were found on much smaller vessels (Extended Data Fig. 3-2*D*). We also evaluated Atp13a5-tdTomato reporter expression in the ovary because this was the only non-CNS tissue with appreciable *Atp13a5* mRNA expression (Extended Data Fig. 1-1*F*). While abundant *Pdgfrb*-EGFP-expressing mural cells are present in the ovary, *Atp13a5*^tdT+^ cells are very rare and require much higher exposure time to detect compared with brain pericytes (Extended Data Fig. 3-2*E*).

**Figure 4.**
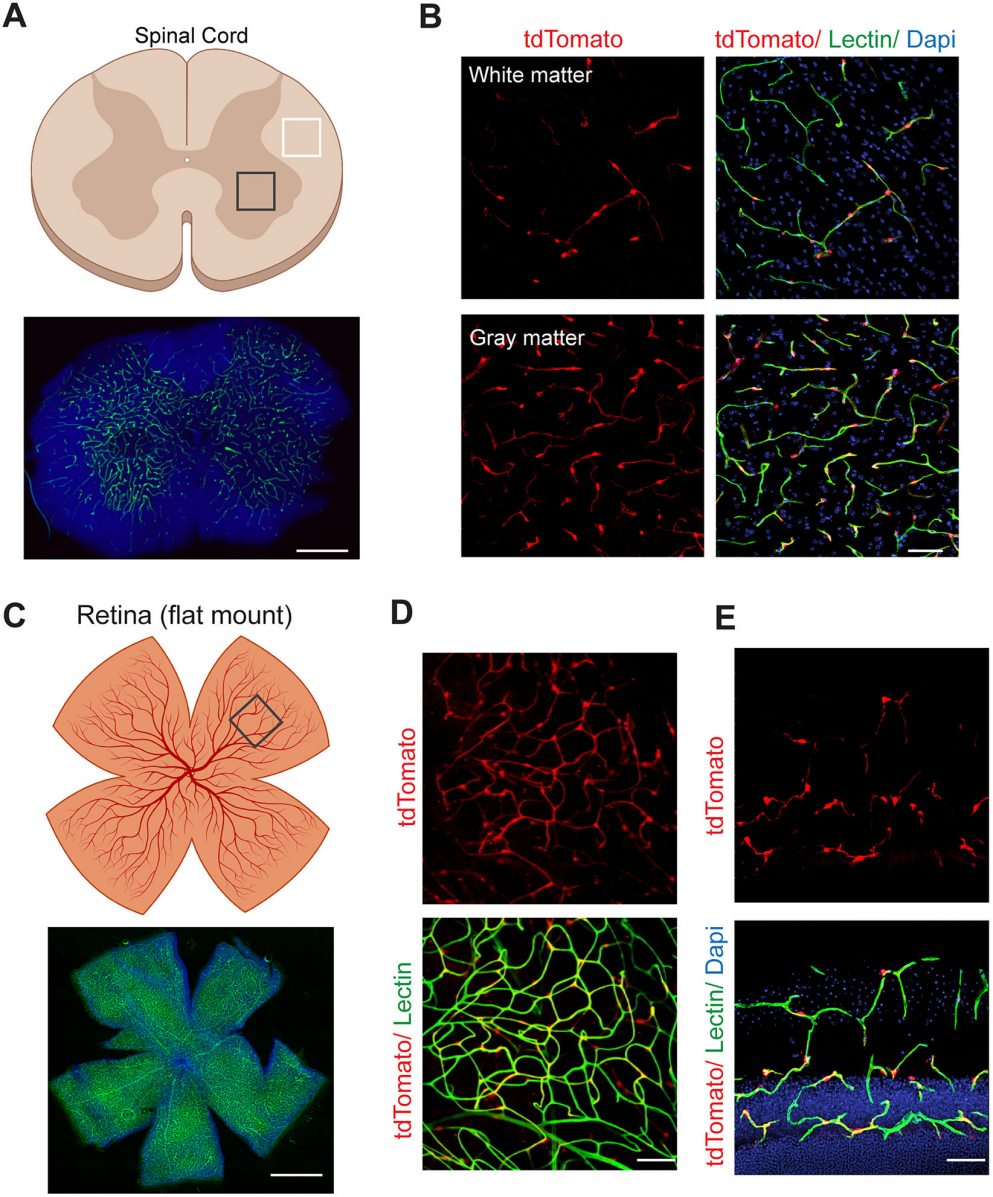
*Atp13a5-driven tdTomato* reporter expression in spinal cord and retina. ***A***, A diagram showing a cross section of mouse spinal cord on the top and lectin angiogram (green) on the bottom. Dapi, nuclear staining. Scale bar, 500 µm. ***B***, tdTomato reporter expression in the spinal cord pericytes of *Atp13a5-2A-CreERT2-IRES-tdTomato* knock-in mice. Scale bar, 50 µm. ***C***, A diagram showing a flat mount preparation of mouse retina on top, and lectin angiogram (green) on bottom. Dapi, nuclear staining. Scale bar, 1 mm. ***D***, ***E***, tdTomato reporter expression in the retinal pericytes of *Atp13a5-2A-CreERT2-IRES-tdTomato* knock-in mice. Scale bar, 50 µm. ***D***, flat mount; ***E***, cross section.

To verify the expression of *Atp13a5* marker throughout the CNS, we also examined the spinal cord and retina in *Atp13a5-2A-CreERT2-IRES-tdTomato* model. We found robust tdTomato signals in both white and gray matter of the spinal cord (Fig. 4*A*,*B*), with a little higher coverage in gray matter. In the retina, pericytes expressing *Atp13a5-*tdTomato were found in most of the microvessels (Fig. 4*C*,*D*), particularly in the ganglion cell layer and inner nuclear layer (Fig. 4*E*), where the blood–retinal barrier exists. Hence, *Atp13a5*-expressing cells uniquely represent mouse CNS pericytes associated with the BBB, blood–spinal cord barrier, and blood–retinal barrier.

### Characterization of the *Atp13a5*-CreER recombinase activity

**Figure 5.**
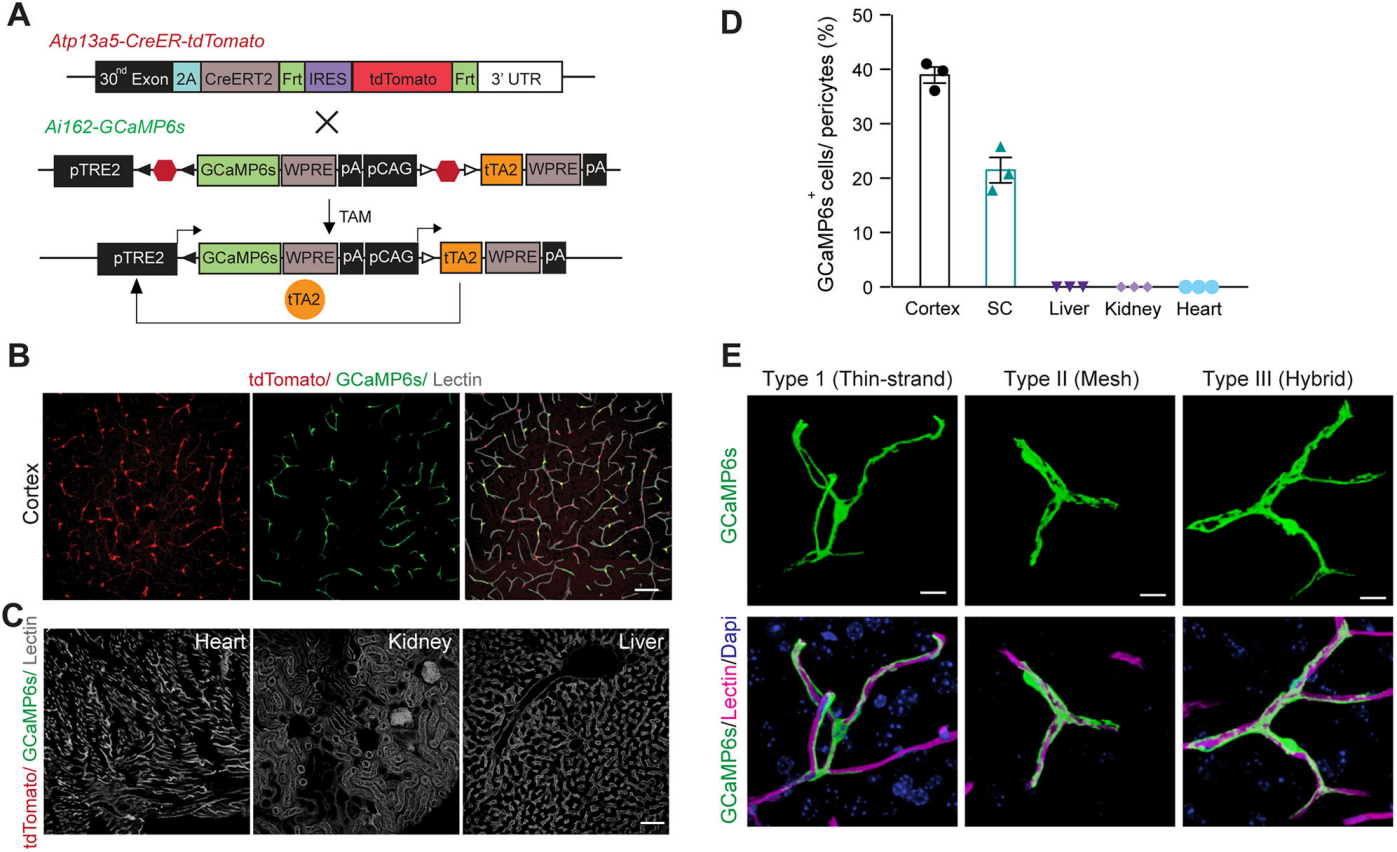
Characterizing the *CreER* recombinase activity. ***A***, Schematic diagram showing the breeding strategy for generating *Atp13a5-2A-CreERT2-IRES-tdTomato; Ai162* mice. ***B***, Representative confocal images of cortical section from a *Atp13a5-2A-CreERT2-IRES-tdTomato; Ai162* mouse, with *Atp13a5*-driven tdTomato (red), tamoxifen-induced GCaMP6s (green), and lectin-labeled endothelial profiles (gray). Scale bar, 50 µm. ***C***, Representative confocal images of heart, kidney, and liver sections from a *Atp13a5-2A-CreERT2-IRES-tdTomato; Ai162* mouse, showing no tdTomato or GCaMP6s expression. Lectin (gray): endothelial profiles. Scale bar, 50 µm. ***D***, Quantification of the percentages of tdTomato^+^ and GCaMP6s^+^ double-positive cells in pericytes in different organs as indicated. *n* = 3 mice. Data are presented in mean ± SEM. ***E***, High-resolution 3D reconstruction of sparse labeled tdTomato^+^ and GCaMP6s^+^ double-positive brain pericytes. Type I, thin-strand pericytes; type II, mesh pericytes; type III, hybrid pericytes. Scale bar, 10 µm. Additional data are provided in Extended Data Figure 5-1.

To test the CreER activity in this *Atp13a5-2A-CreERT2-IRES-tdTomato* model, we crossed it with the Cre-dependent and Tet-controllable Ai162 line with fluorescent calcium-indicator GCaMP6s (Daigle et al., 2018) and induced the CreER activity with tamoxifen administration (Fig. 5*A*; also see Materials and Methods). With four injections of tamoxifen at 40 mg/kg (Nikolakopoulou et al., 2019), we observed nearly 40% of tdTomato^+^ brain pericytes expressing robust GCaMP6s protein (Fig. 5*B*), but not in peripheral tissues such as the heart, kidney, or liver (Fig. 5*C*,*D*). This sparse labeling also allows us to clearly resolve the morphology of single pericytes. While the majority of the tdTomato^+^ and GCaMP6s^+^ double-positive profiles exhibit elongated processes covering the microvessels (Type I, 46 ± 2%), we also observed pericytes with shorter processes wrapping around the whole microvessel (Type II, 21 ± 3%), as well as a hybrid type with both elongated and wrapping processes (Type III, 33 ± 2%; Fig. 5*E*, Extended Data Fig. 5-1*A,B*). Type I and type II are known as thin-strand and mesh pericytes, respectively (Berthiaume et al., 2018), and the hybrid type may represent a transition between them, which exhibits differences in length and branch numbers based on the measurement of main branches (Extended Data Fig. 5-1*C,D*), suggesting the heterogeneity of CNS pericytes remains to be explored. The recombination efficiency can be improved with increased tamoxifen treatment, e.g., after seven injections nearly 80% of tdTomato^+^ brain pericytes expressed GCaMP6s protein (Extended Data Fig. 5-1*E,F*). Taken together, our data demonstrated that the new *Atp13a5* marker reveals the CNS pericytes, and *Atp13a5-2A-CreERT2-IRES-tdTomato* model is a reliable tool with its reporter and Cre recombinase dual activities to explore the biology of CNS pericytes in vivo.

10.1523/JNEUROSCI.0727-24.2024.f5-1 Figure 5-1 **Additional characterization of the CreER recombinase activity**. (**A**) Additional 3D reconstruction of sparse labelled *Atp13a5*-GCaMP6s^+^ brain pericytes, showing the typical thin-strand, mesh and hybrid morphologies. Scale bar: 10  µm. (**B**) Pie chart showing the percentage distribution of three different brain pericyte types based on quantification of *Atp13a5*-GCaMP6s^+^ brain pericytes from 5 individual mice. (**C-D**) Quantifications of the pericyte length based on the main branches (C), and average number of main branches in each pericytes. Data are presented in mean ± SEM; n = 34-39 cells. (**E**) Representative confocal images of cortical section from *Atp13a5-tdTomato; Ai162* mice received 7 doses of 40  mg/kg tamoxifen. Images in the bottom row showing boxed regions with a venule (outlined by dash lines) without GCaMP6 or tdTomato expression. Bar: 100  µm. (**F**) Quantification of GCaMP6-positive cells in tdTomato-positive pericytes as an indication of CreERT2 efficiency. N = 3 mice per group. Download Figure 5-1, TIF file.

### Developmental regulation of *Atp13a5* marker

**Figure 6.**
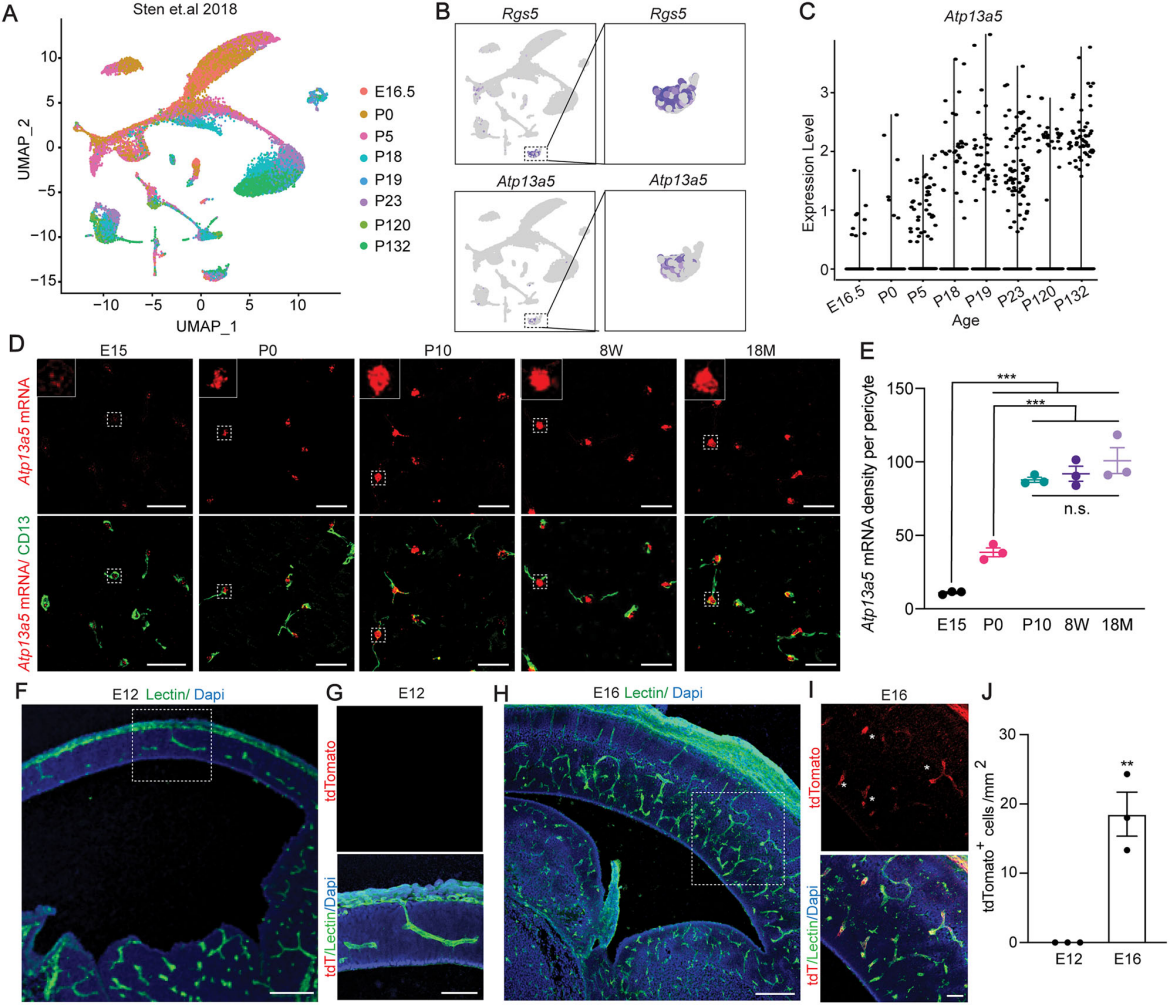
Developmental regulation of *Atp13a5* marker. ***A***, UMAP of 24,185 mouse brain cells from multiple ages. Each dot was color-coded and annotated by different ages. ***B***, UMAP plots showing scRNA-seq data, colored by gene expression value, showing *Rgs5* and *Atp13a5* expression. ***C***, Violin plots showing *Atp13a5* expression in different ages. ***D***, Representative images for *Atp13a5* mRNA expression and CD13 in brain sections from different ages. Scale bar, 50 µm. Sections: 15 µm thick. ***E***, *Atp13a5* expression density per pericyte in various developmental stages. Data are presented in mean ± SEM; *n* = 3 mice per age group; ***p < 0.001, n.s., no significant difference, by one-way ANOVA with Tukey’s test. ***F***, A representative image of an *Atp13a5-2A-CreERT2-IRES-tdTomato* embryonic cortex at E12. Scale bar, 200 µm. Sections: 30 µm thick. ***G***, High magnification of boxed region in ***F***. tdTomato was not detected in the cortex at E12. Scale bar, 100 µm. ***H***, A representative image of an *Atp13a5-2A-CreERT2-IRES-tdTomato* embryonic cortex at E16. Scale bar, 200 µm. Sections: 30 µm thick. ***I***, High magnification of boxed region in ***H***. tdTomato can be detected in the cortex at E16. Scale bar, 50 µm. Sections: 30 µm thick. ***J***, Quantification of tdTomato^+^ pericyte numbers per mm^2^ in E12 and E16 mouse brain. *n* = 3 mice. **p < 0.01 by Student's *t* test. Data are presented in mean ± SEM. Additional data are provided in Extended Data Figure 6-1.

To determine the specification of brain pericytes during development, we analyzed 24,185 single cells from mouse dentate gyrus (Hochgerner et al., 2018), spanning perinatal [embryonic day (E) 16.5 to postnatal day (P) 5], juvenile (P18–P23), and adult (P120–P132) ages (Fig. 6*A*). Again, *Atp13a5-*expressing cells are only present in the pericyte cluster (Fig. 6*B*, Extended Data Fig. 6-1*A*). Interestingly, the transcript of *Atp13a5* expression increased from E16.5 to birth and P18 and was sustained throughout adulthood (Fig. 6*C*). We further performed FISH with RNAscope probes at different ages. By quantifying the density of *Atp13a5* transcripts in CD13^+^ pericytes, we found that *Atp13a5* was expressed in ∼10% CD13^+^ brain pericytes at E15 (Fig. 6*D*), <50% at birth (P0), while at P10, most brain pericytes expressed *Atp13a5* the same as 8 weeks (8W) and 18 months (18M; Fig. 6*E*). The transcriptional changes of *Atp13a5* were recapitulated by the *Atp13a5* transgenic reporter: tdTomato^+^ brain pericytes were not seen at E12 (Fig. 6*F*,*G*), barely visible at E15 (Extended Data Fig. 6-1*B,C*), and became prominent at E16 (Fig. 6*H–J*). At P0, the tdTomato profiles were seen on many developing vessels (Extended Data Fig. 6-1*D,E*). This embryonic developmental period is also critical for BBB development (Zhao et al., 2015), suggesting that brain pericyte's specification is part of the BBB establishment.

10.1523/JNEUROSCI.0727-24.2024.f6-1 Figure 6-1 **Developmental regulation of Atp13a5 driven tdTomato reporter expression.** (**A**) UMAP plots showing scRNA-seq data, colored by gene expression value, showing *Pdgfrb* and *Kcnj8* expression, based on dataset GSE95753. (**B**) A representative image of *Atp13a5-2A-CreERT2-IRES-tdTomato* mouse cortex at E15. Scale bar: 200  µm. Sections: 30  µm thick. (**C**) High magnification of boxed region in B. Asterisks: weak tdTomato signals detected in cortex at E15. Scale bar: 100  µm. (**D**) A representative image of *Atp13a5-2A-CreERT2-IRES-tdTomato* mice brain at P0. Scale bar: 100  µm. Sections: 35  µm thick. (**E**) High magnification of boxed region in D. Strong tdTomato signals can be detected in cortex at P0. Scale bar: 50  µm. Sections: 35  µm thick. Download Figure 6-1, TIF file.

## Discussion

Brain pericytes regulate vascular development and microvascular functions; however, their identification still requires a combination of inadequate markers shared with other cell types, including closely related fibroblasts and VSMCs. This has become a major hurdle to a better understanding of pericyte biology, heterogeneity, and contributions toward human diseases. Here, we identified a novel genetic marker *Atp13a5* for mouse brain pericytes through multiple transcriptomic datasets and generated a new transgenic tool based on this allele. This *Atp13a5* genetic marker, as well as the *Atp13a5-2A-CreERT2-IRES-tdTomato* knock-in reporter model, demonstrated high specificity to CNS pericytes. First, *Atp13a5* is not expressed in VSMCs or perivascular fibroblasts; second, it exclusively labels CNS pericytes, but not the peripheral ones. More importantly, when tracing the *Atp13a5*-expressing cells in the reporter model, we surprisingly found that they are exclusively located in the CNS regions including the brain, spinal cord, and retina, while the pericytes in the peripheral tissues do not express this marker, e.g., heart, kidney, and liver. Therefore, the *Atp13a5* marker represents a key molecular feature of the CNS pericytes, separating them from the peripheral ones, which makes the *Atp13a5-2A-CreERT2-IRES-tdTomato* knock-in model as a useful tool for studying CNS pericytes. While *Atp13a5*-tdTomato is not expressed on large vessels, we have not ruled out its presence on postcapillary venules and precapillary arterioles. Future studies are still needed to establish *Atp13a5*-pericyte profiles on mural cell zonation along the brain vascular tree at different branching orders and validate it as a marker for small vessels in development and pathophysiological conditions.

The mammalian BBB is central to the CNS health and functions, which is established during embryonic development and influenced by the neural environment (Zhao et al., 2015). The developmental origin of pericytes seems to be rather complex (Yamazaki and Mukouyama, 2018); and their heterogeneity has only been confirmed between central and peripheral tissues, but not within the CNS (Vanlandewijck et al., 2018). The finding of *Atp13a5*-based brain pericyte heterogeneity now allows us to examine brain pericytes development and the fate determination of BBB pericytes more accurately, at anatomical and transcriptional levels. More specifically, we found *Atp13a5* is turned on approximately E15 in mice, when the BBB starts to be functional (Ben-Zvi et al., 2014), which is different from other known markers (Jung et al., 2018). This suggests that brain pericytes can also be influenced by the local neural environment and metabolic reprogramming (Sheikh et al., 2020) and switch their property toward more specialized brain pericytes. They could be regulated by the Wnt signaling and induced by the neuronal activity, just like the BBB endothelium (Zhao et al., 2015), or by completely new mechanism yet to be deciphered. Nevertheless, more comprehensive decoding of the transcriptional differences between the CNS and peripheral pericytes remains to be explored. Perhaps, the double-promotor model combing *Atp13a5-*tdTomato and *Pdgfrb*-EGFP might help, particularly at the embryonic stages when developing brain pericytes are quite heterogeneous.

Although pericytes and VSMCs are anatomically positioned at different locations of the vascular segment and are functionally different, their biological identifies are similar in many ways. This has made it difficult to understand the in vivo functions of brain pericytes and perhaps led to certain disagreements regarding the role of pericytes in regulating cerebral blood flow (Hall et al., 2014; Hill et al., 2015; Hartmann et al., 2021). This issue perhaps can be resolved now with the new *Atp13a5-2A-CreERT2-IRES-tdTomato* knock-in model. It is a versatile tool with not only an endogenous tdTomato reporter for tracing CNS pericytes, as well as a tamoxifen-inducible Cre recombinase for effective genetic manipulation, specifically in these cells. The robustness of the tdTomato signal in this model enables cell sorting for molecular profiling and cell culture without the need for antibodies, as well as intravital imaging in vivo. More importantly, the CreERT2 element will provide us an inducible approach in vivo, including genetic manipulation and ablation, especially in cases when brain VSMCs or peripheral pericytes may confound interpretations. In addition, the IRES-tdTomato sequence is further flanked by two flp recombinase target (frt) sites for future removal of the reporter (Nern et al., 2011).

Last but not the least, pericyte degeneration associated with BBB dysfunction is found in a spectrum of CNS disorders, including AD and other dementia (Sweeney et al., 2016). Therefore, the identification of *Atp13a5* as a specific BBB pericyte marker will advance our studies in vascular contributions to various brain disorders across the lifespan. Beyond the application of the transgenic model, the identification of *Atp13a5* as a CNS pericyte-specific marker can be used to develop other genetic tools, such as ATP13A5-specific antibodies, and new viral vector designs to target BBB pericytes more specifically with artificial promoters (Jüttner et al., 2019).
