# Supplementary figures and images for: Atp13a5 Marker Reveals Pericyte Specification in the Mouse Central Nervous System
Source: J Neurosci. 2024 Sep 11;44(43):e0727242024. doi: 10.1523/JNEUROSCI.0727-24.2024 (PMC11502228; doi:10.1523/JNEUROSCI.0727-24.2024)

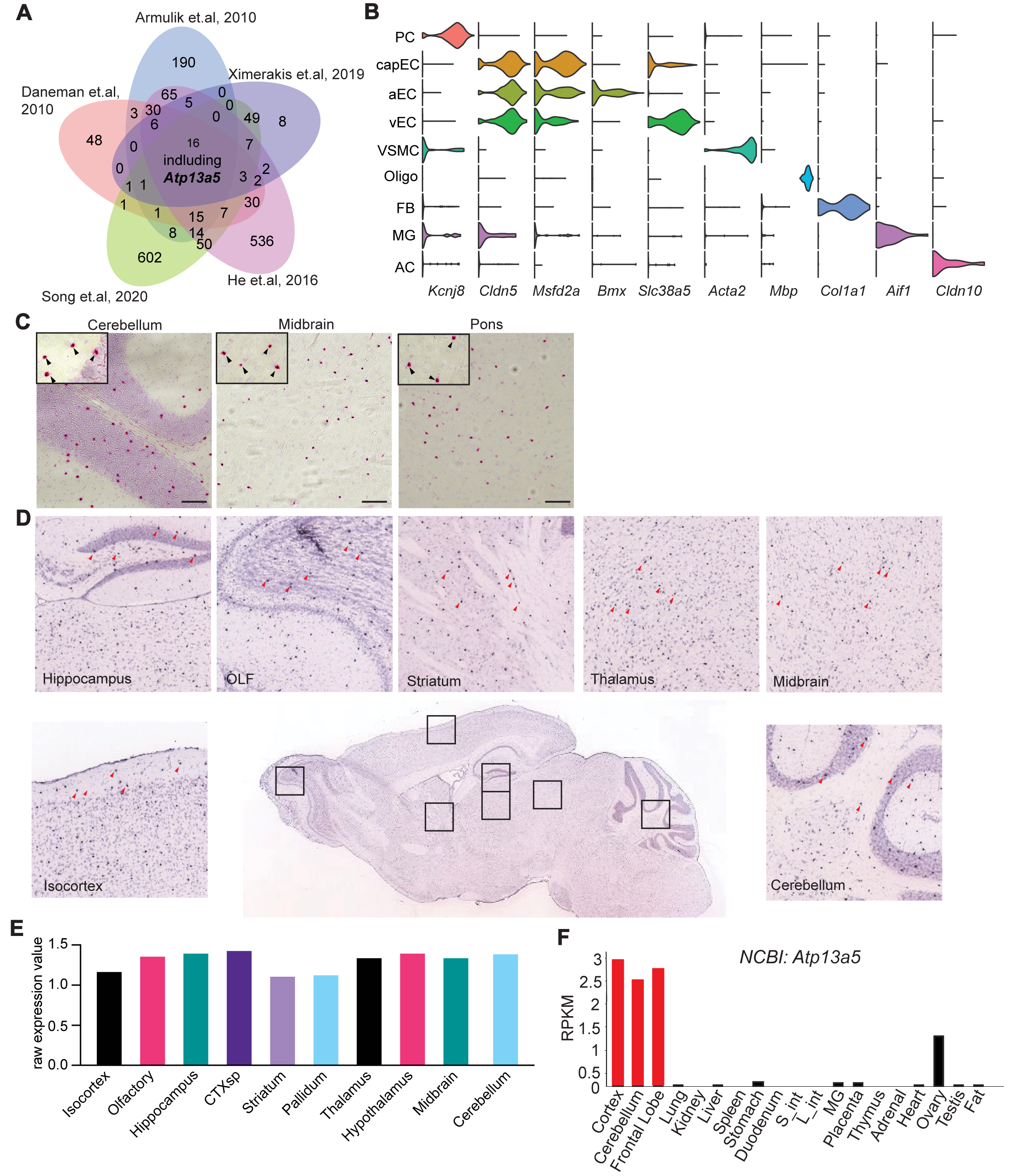

Supplement: Figure 1-1 — Atp13a5 expression in the mouse brain. (A) Venn plot showing the overlaid genes between different datasets. (B) Violin plots showing gene markers that distinguish across vasculature cells. Genes are colored by cell types. PC: Pericytes; capEC: capillary endothelial cells; aEC: arterial endothelial cells; vEC: venous endothelial cells; VSMC: vascular smooth muscle cells; Oligo: Oligodendrocytes; FB: Fibroblast; MG: microglia; AC: astrocytes. (C) Representative images for Atp13a5 mRNA expression (Red) in various mouse brain region. Scale bar: 100 µm. Sections: 10 µm thick. (D) ISH of Atp13a5 mRNA expression in various mouse brain region from Allen Brain Atlas. (E) Raw expression value of Atp13a5 mRNA expression in various mouse brain region. ISH expression data are from Allen Brain Atlas obtained from 56 days old adult male C57BL/6J mice (available from: http://mouse.brain-map.org). OLF, olfactory bulb; CTXsp, cortex subplate. (F) Bar plot showing Atp13a5 expression pattern in NCBI dataset. Red bar indicated the brain tissue. L_int: large intestine; S_int: small intestine; MG: mammary gland. Download Figure 1-1, TIF file. [file jneuro-44-e0727242024-s001.tif]

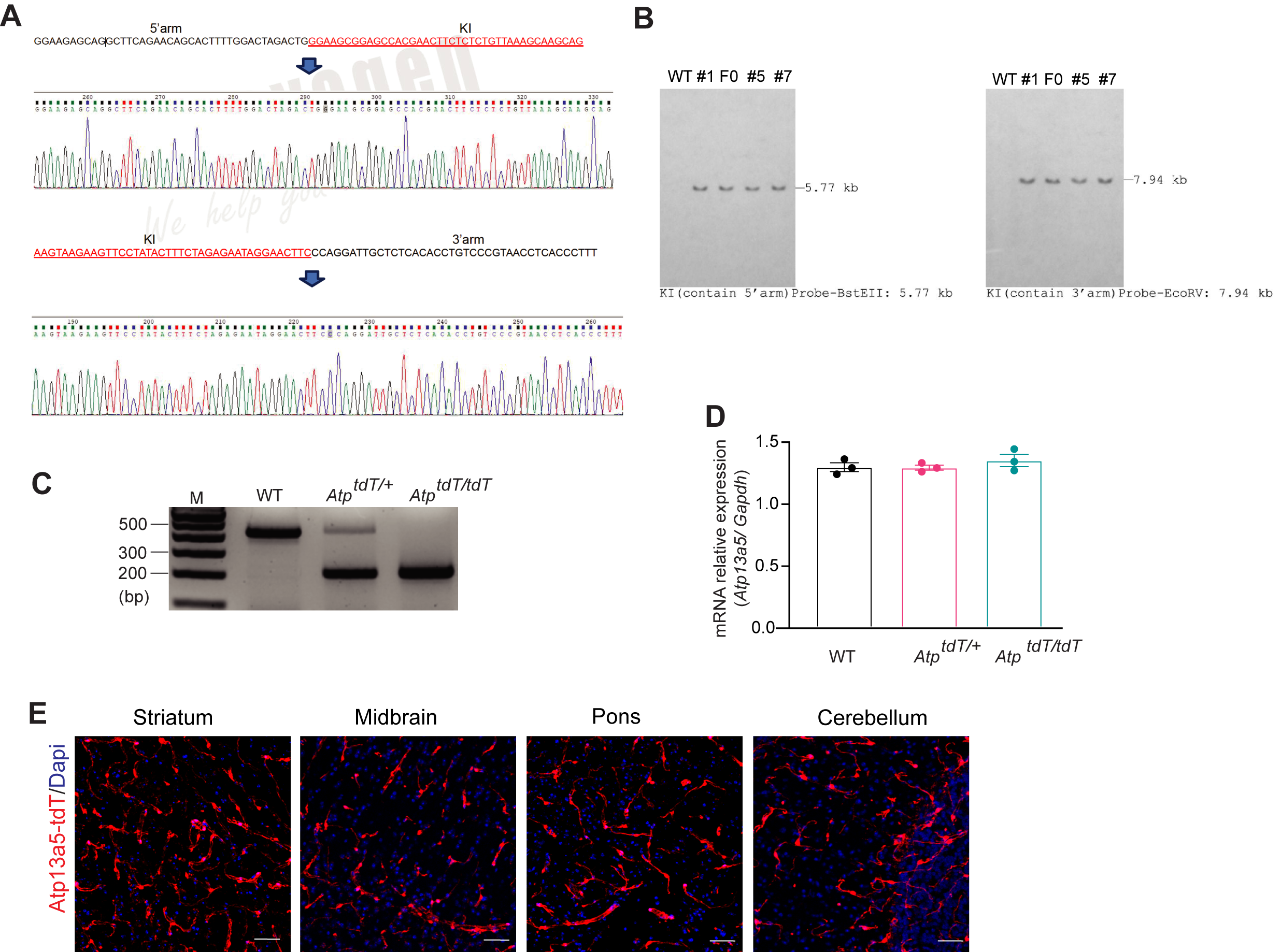

Supplement: Figure 2-1 — Generation and validation of the Atp13a5-2A-CreERT2-IRES-tdTomato model. (A) Sequencing analysis of F1 AtptdT/+ mice showing the insertions at 5’arm (top) and 3’arm (bottom). No additional mutation or deletion were found. (B) Southern blotting analysis showing F0 and 3 F1 founders (#1, #5, #7) carrying the intact allele based on hybridization of probes targeting the 5’ and 3’ arms, compared to a WT littermate. (C) Genotyping result showing the genotype of WT (with a 432-bp band), AtptdT/+ (with 432-bp and 212-bp bands) and AtptdT/tdT (with a 212-bp band). Atp, Atp13a5; tdT, tdTomato. (D) The endogenous gene expression of Atp13a5 relative to Gapdh in brains from 8-week-old WT, AtptdT/+ and AtptdT/tdT mice (n = 3 mice each). Data are presented in mean ± SEM. Download Figure 2-1, TIF file. [file jneuro-44-e0727242024-s002.tif]

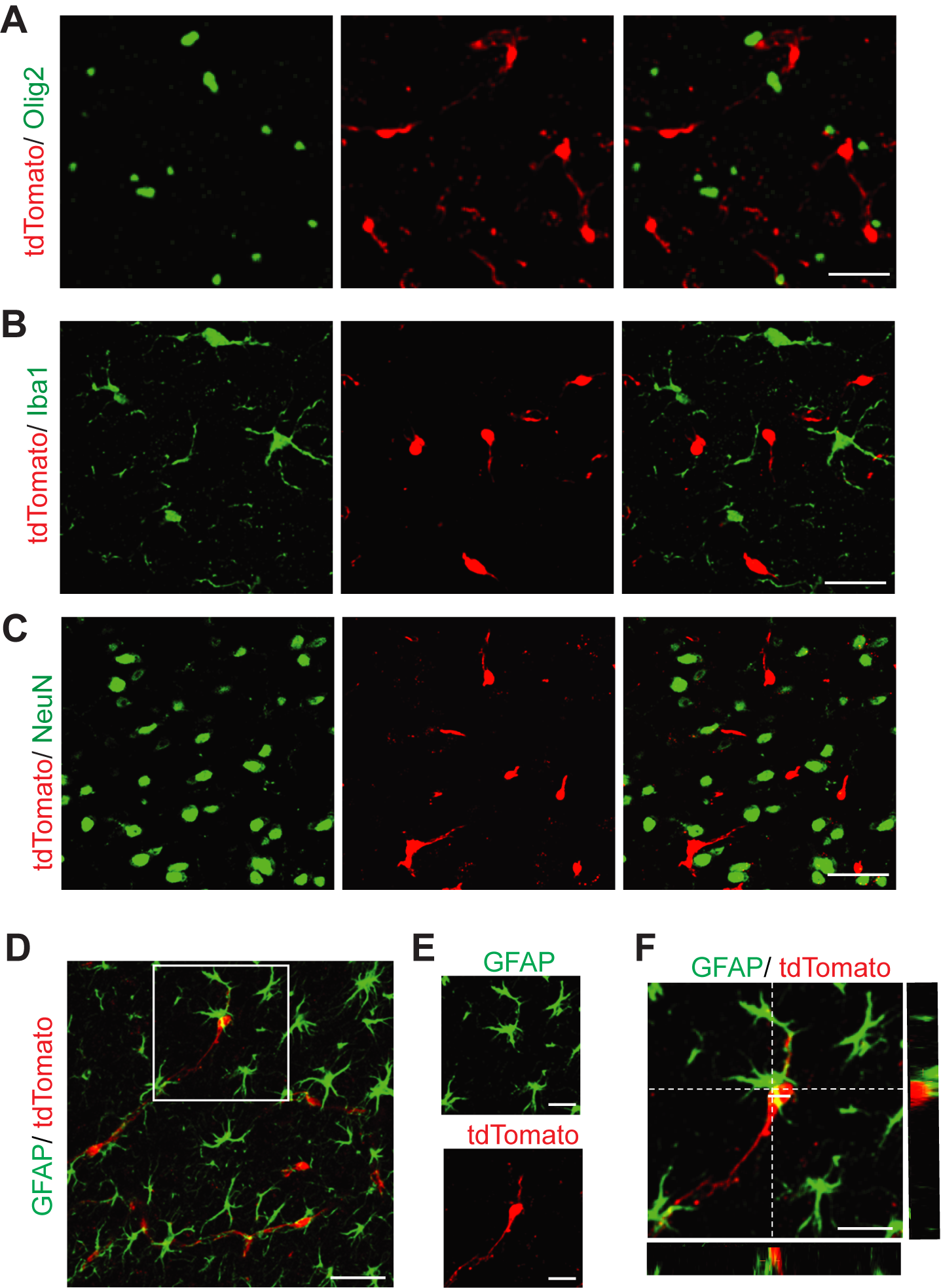

Supplement: Figure 3-1 — Characterization of Atp13a5-2A-CreERT2-IRES-tdTomato mouse brain. (A) Representative images for Atp13a5-tdT reporter expression in mouse striatum, midbrain, pons and cerebellum regions. (B-E) Representative confocal images showing that tdTomato is not expressed in Olig2+ Oligodendrocytes (B), ionized calcium binding adaptor molecule 1 (Iba1)+ microglia (C), NeuN+ cortical neurons (D), and glial fibrillar acidic protein (GFAP)+ astrocytes (E). High magnification of boxed region in E is shown in F, and orthogonal view is shown in G. A-E: Scale bar: 50 µm. F and G: Scale bar: 25 µm. Download Figure 3-1, TIF file. [file jneuro-44-e0727242024-s003.tif]

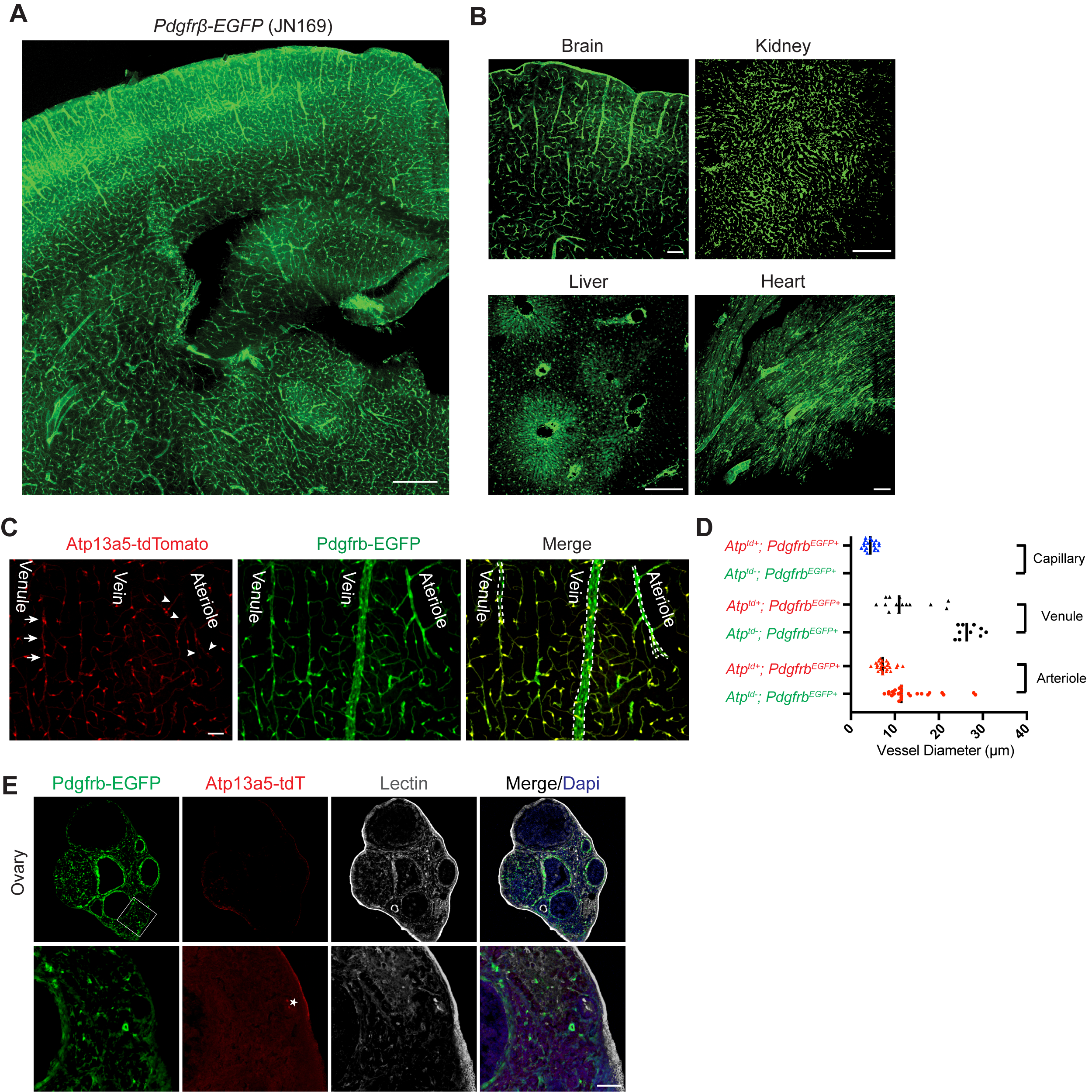

Supplement: Figure 3-2 — EGFP reporter expression in Pdgfrb-EGFP mouse. (A) A representative image of Pdgfrb-EGFP mouse brain. Scale bar: 200 µm. Sections: 35 µm thick. (B) EGFP reporter expression in brain (cortex) and peripheral tissues such as kidney, liver and heart. Scale bar: 100 µm. Sections: 35 µm thick. (C-E) Characterization of Atp13a5-tdTomato; Pdgfrb-EGFP double reporter mice. (C) Representative confocal images of cortical section from Atp13a5-tdTomato; Pdgfrb-EGFP mice. Bar = 30 µm. (D) Quantification of diameters of capillary, postcapillary venules and precapillary arterioles with Atp13a5tdT+ and PdgfrbEGFP+ pericytes, or Atp13a5tdT- and PdgfrbEGFP+ mural cells. (E) Representative confocal images of ovary section from Atp13a5-tdTomato; Pdgfrb-EGFP mice, using the same imaging setting as in C. Bar = 50 µm. Boxed region is re-scanned with increased exposure, and shown in the bottom. Download Figure 3-2, TIF file. [file jneuro-44-e0727242024-s004.tif]

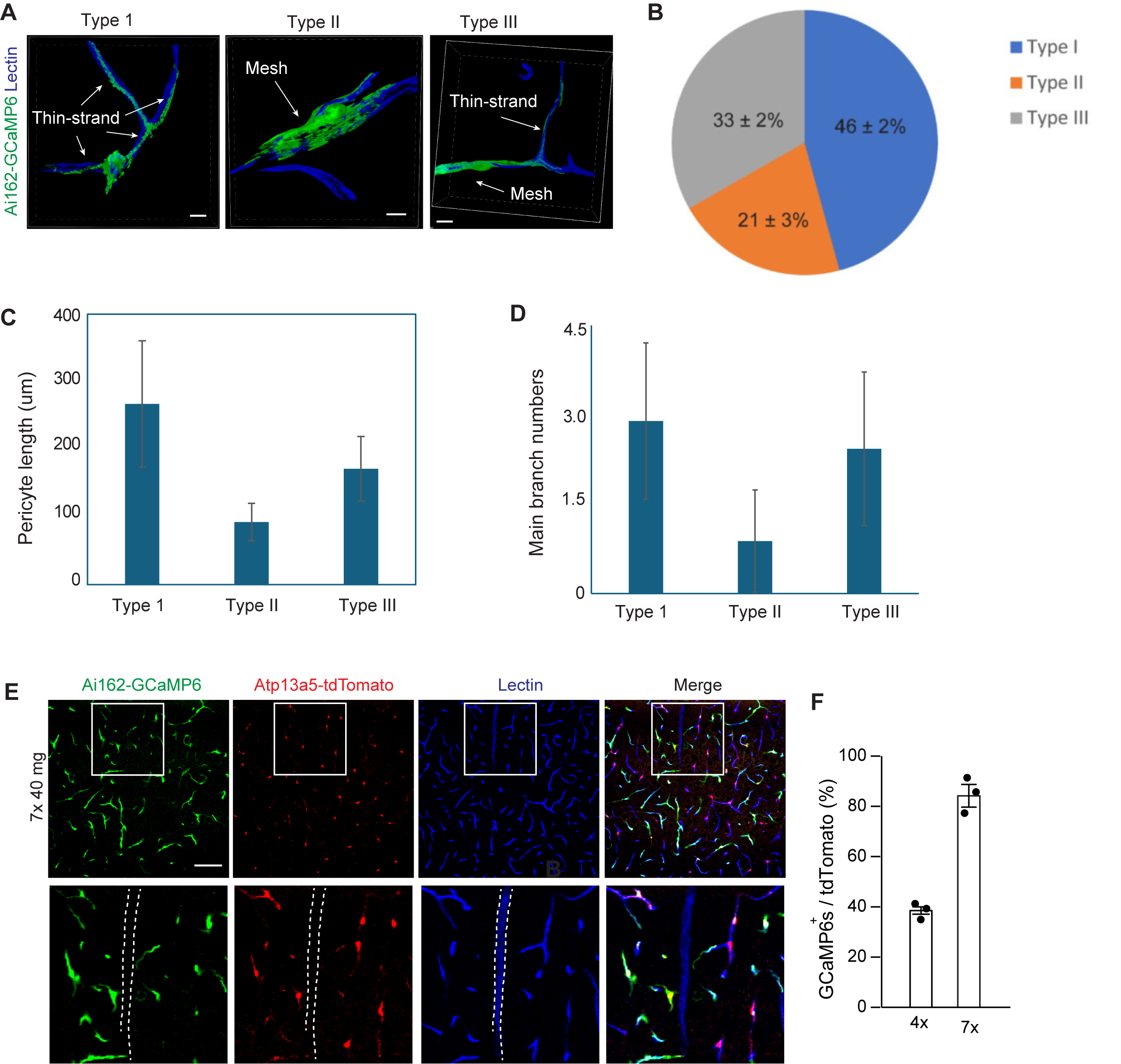

Supplement: Figure 5-1 — Additional characterization of the CreER recombinase activity. (A) Additional 3D reconstruction of sparse labelled Atp13a5-GCaMP6s+ brain pericytes, showing the typical thin-strand, mesh and hybrid morphologies. Scale bar: 10 µm. (B) Pie chart showing the percentage distribution of three different brain pericyte types based on quantification of Atp13a5-GCaMP6s+ brain pericytes from 5 individual mice. (C-D) Quantifications of the pericyte length based on the main branches (C), and average number of main branches in each pericytes. Data are presented in mean ± SEM; n = 34-39 cells. (E) Representative confocal images of cortical section from Atp13a5-tdTomato; Ai162 mice received 7 doses of 40 mg/kg tamoxifen. Images in the bottom row showing boxed regions with a venule (outlined by dash lines) without GCaMP6 or tdTomato expression. Bar: 100 µm. (F) Quantification of GCaMP6-positive cells in tdTomato-positive pericytes as an indication of CreERT2 efficiency. N = 3 mice per group. Download Figure 5-1, TIF file. [file jneuro-44-e0727242024-s008.tif]

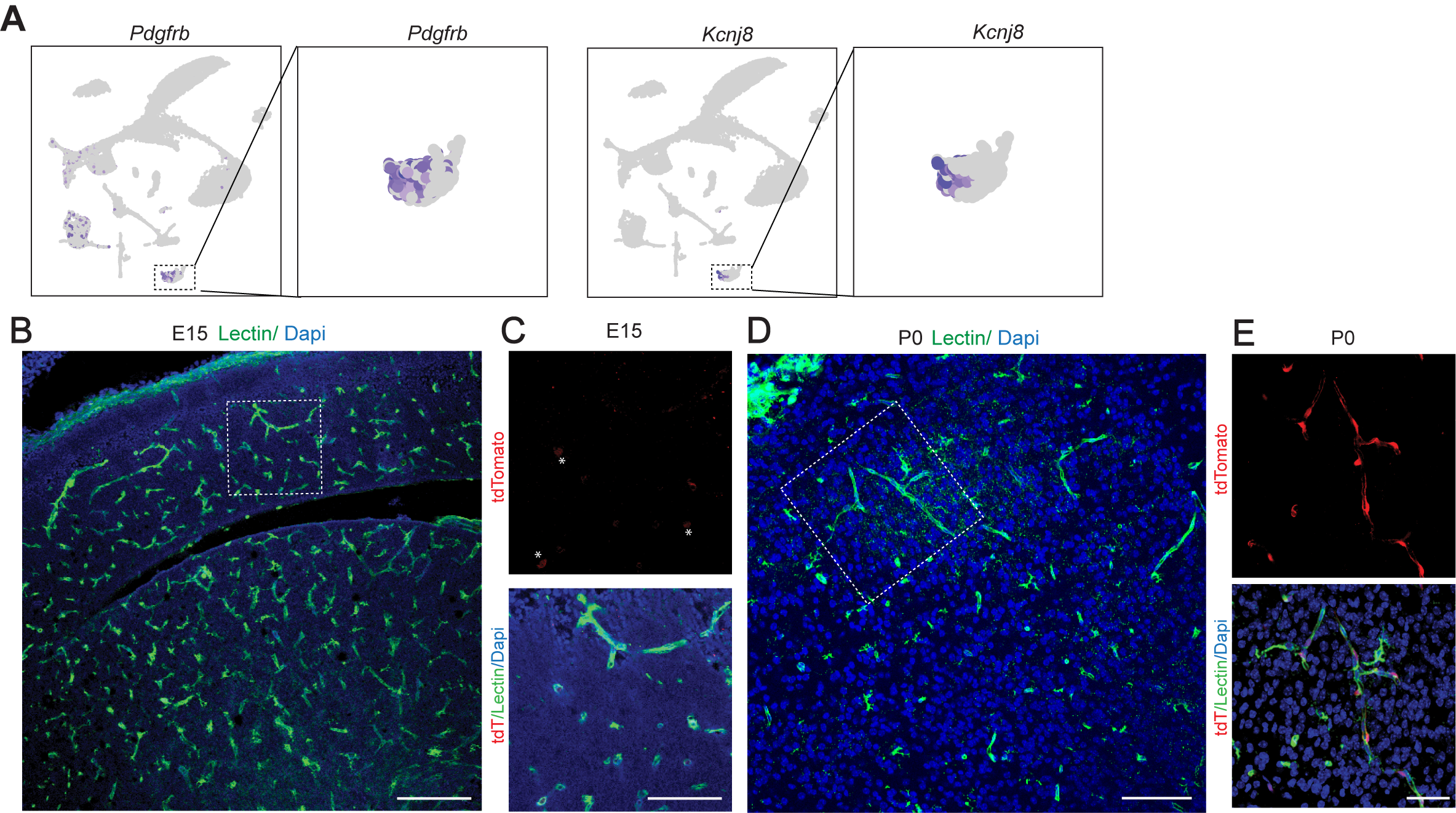

Supplement: Figure 6-1 — Developmental regulation of Atp13a5 driven tdTomato reporter expression. (A) UMAP plots showing scRNA-seq data, colored by gene expression value, showing Pdgfrb and Kcnj8 expression, based on dataset GSE95753. (B) A representative image of Atp13a5-2A-CreERT2-IRES-tdTomato mouse cortex at E15. Scale bar: 200 µm. Sections: 30 µm thick. (C) High magnification of boxed region in B. Asterisks: weak tdTomato signals detected in cortex at E15. Scale bar: 100 µm. (D) A representative image of Atp13a5-2A-CreERT2-IRES-tdTomato mice brain at P0. Scale bar: 100 µm. Sections: 35 µm thick. (E) High magnification of boxed region in D. Strong tdTomato signals can be detected in cortex at P0. Scale bar: 50 µm. Sections: 35 µm thick. Download Figure 6-1, TIF file. [file jneuro-44-e0727242024-s005.tif]
